# iMGEins: detecting novel mobile genetic elements inserted in individual genomes

**DOI:** 10.1186/s12864-018-5290-9

**Published:** 2018-12-18

**Authors:** Junwoo Bae, Kyeong Won Lee, Mohammad Nazrul Islam, Hyung-Soon Yim, Heejin Park, Mina Rho

**Affiliations:** 1 Department of Electronics and Computer Engineering, Hanyang University, Seoul, Korea; 2 Marine Biotechnology Research Center, Korea Institute of Ocean Science and Technology, Ansan, Korea; 3 Department of Marine Biotechnology, Korea University of Science and Technology, Daejeon, Korea; 4 Department of Biotechnology, Sher-e-Bangla Agricultural University, Dhaka, 1207 Bangladesh; 5 Department of Computer Science and Engineering, Hanyang University, Seoul, Korea; 6 Department of Biomedical Informatics, Hanyang University, Seoul, Korea

**Keywords:** Mobile genetic elements, Paired-end sequencing, Long insertions, Structural variations

## Abstract

**Background:**

Recent advances in sequencing technology have allowed us to investigate personal genomes to find structural variations, which have been studied extensively to identify their association with the physiology of diseases such as cancer. In particular, mobile genetic elements (MGEs) are one of the major constituents of the human genomes, and cause genome instability by insertion, mutation, and rearrangement.

**Result:**

We have developed a new program, iMGEins, to identify such novel MGEs by using sequencing reads of individual genomes, and to explore the breakpoints with the supporting reads and MGEs detected. iMGEins is the first MGE detection program that integrates three algorithmic components: discordant read-pair mapping, split-read mapping, and insertion sequence assembly. Our evaluation results showed its outstanding performance in detecting novel MGEs from simulated genomes, as well as real personal genomes. In detail, the average recall and precision rates of iMGEins are 96.67 and 100%, respectively, which are the highest among the programs compared. In the testing with real human genomes of the NA12878 sample, iMGEins shows the highest accuracy in detecting MGEs within 20 bp proximity of the breakpoints annotated.

**Conclusion:**

In order to study the dynamics of MGEs in individual genomes, iMGEins was developed to accurately detect breakpoints and report inserted MGEs. Compared with other programs, iMGEins has valuable features of identifying novel MGEs and assembling the MGEs inserted.

**Electronic supplementary material:**

The online version of this article (10.1186/s12864-018-5290-9) contains supplementary material, which is available to authorized users.

## Background

Mobile genetic elements (MGEs) constitute a significant portion of the eukaryotic genomes, and play important roles as a driver of genomic instability and regulatory elements [1–8]. As such, the identification of novel MGEs in individual genomes and the analysis of their dynamics are important for a better understanding of the genome instability as one of the factors responsible for diseases. While several programs including BreakDancer [9] and Pindel [10] are currently used in order to call structural variations, such as single nucleotide polymorphisms (SNPs), translocations, tandem duplications, and relatively small indels, they rarely detect large insertions such as MGEs.

Based on the transposition mechanism, MGEs are classified to two different groups: DNA transposons and retrotransposons. Retrotransposons are further classified into LTR retrotransposons and non-LTR retrotransposons. Detecting all types of MGEs and large novel insertions is still very challenging. As diverse species are sequenced and comparative genomics are actively applied, finding novel MGEs is becoming an important subject in genome studies. A systematic approach is thus needed to identify novel MGEs in individual genomes by using high-throughput sequencing reads. Over the past decade, several programs have become available that can search MGEs with whole genome sequencing reads [11–40]. Notable examples include alu-detect [12], RetroSeq [13], Tangram [14], TraFiC [40], TranspoSeq [29], Tea [22], TEMP [31], Mobster [24], nsg_te_mapper [37], PopoolationTE [38], and MELT [39]. In addition, there is an integrated pipeline, McClintock [41], which runs multiple MGE detection programs.

Typically, MGE detection with whole genome paired-end sequencing data is realized by two approaches: discordant read-pair mapping and split-read mapping. In addition, contig assembly also can be used to find the novel insertion including MGEs. After assembling MGEs or novel insertions, iMGEins finds a pair of reads in which one end are aligned on the MGEs and the other are aligned around the breakpoint to connect them together. Most of the programs use discordant read-pair mapping to infer the positions where the fragments are inserted [13, 14, 20, 24, 31, 39]. Specifically, this approach utilizes read-pair mapping information, so that one-end read maps uniquely to reference genome while the mated read maps to the MGE library. However, discordant read-pair mapping cannot find the exact coordinates of breakpoints, since discordant read at one side implies that it is not aligned on the reference genome (Additional file 1: Figure S1). Moreover, such method cannot find novel insertions that are not included in the library. These shortcomings are overcome by certain programs. Among the six programs discussed above [13, 14, 20, 24, 31, 39], Tangram [14], ITIS [20], Mobster [24], TEMP [31], and MELT [39] employ split-read mapping for the breakpoints identified by discordant read-pair mapping information.

In this paper, we report a comprehensive MGE detection program, iMGEins, which combines two approaches described above. iMGEins can detect novel MGE insertions by using sequencing reads of individual genomes, and annotate MGEs based on the sequence homology. In addition, we provide a de novo assembly mode to identify novel MGEs inserted differently to the individual genomes. We have compared iMGEins with RetroSeq [13], TEMP [31], PopoolationTE [38], and MELT [39] by using a set of simulated reads from modified human genomes. Our experiments showed that: (i) the average recall rate is 96.67% and precision rate is 100%, which is the highest among the programs compared. Using the NA12878 dataset, we have also compared the performance of iMGEins with five existing programs to find that iMGEins can detect with highest accuracy the MGEs within 20 bp proximity of the breakpoints annotated. Additionally, we demonstrated that iMGEins could locate the chimeric points in assembled genomes.

## Implementation

iMGEins predicts breakpoints where fragments of different sizes are inserted, and annotates such inserted fragments to find novel MGEs by using read and contig information. The program processes the data in four consecutive steps: 1) classifying reads; 2) predicting breakpoints; 3) identifying MGEs by using one-end unmapped reads; 4) assembling MGEs (Fig. 2).

### Read classification using mapping information

In the first step, iMGEins uses the sequence alignment map (SAM) format file to search for soft-clipped (i.e. partially aligned) or discordant reads around the breakpoints (Fig. 2a). The current version of iMGEins has been tested with alignments from Bowtie2 [42], BWA [43], and Mosaik [44]. As the default mapping program, Bowtie2 was used with the ‘--local-sensitive’ option to allow local alignments for soft-clipped reads.

According to the mapping status, such as bitwise FLAG and the CIGAR string in the SAM format file, the reads are grouped into one of the following three types:

- Soft-clipped (S) if the reads are partially mapped with the sufficient length of clipped sequence.
- Mapped (M) if one-end read is fully mapped.
- Unmapped (U) if one-end reads is not mapped.

**Fig. 1:**
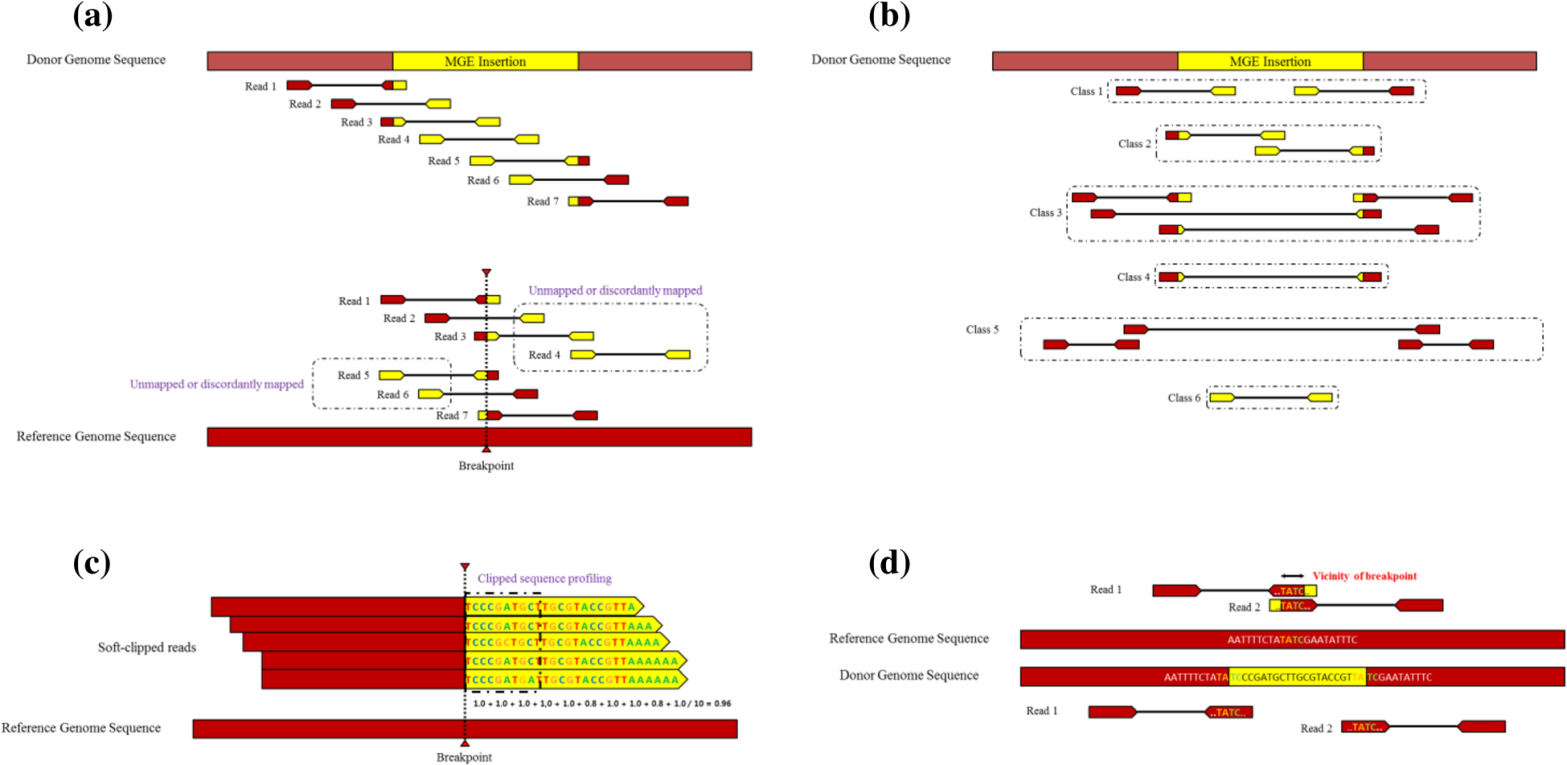
Classification of reads to find and annotate MGEs. **a** When reads are aligned with local alignment, there exist three types of reads: reads are mapped (M, Reads 1, 2, 6, and 7); reads spanning MGE insertion are soft-clipped (S, Reads 1, 3, 5, and 7); reads from MGE insertions are unmapped (U, Reads 2, 3, 4, 5, and 6). **b** All reads are grouped into six classes: class 1 (M-U or U-M), class 2 (S-U or U-S), class 3 (M-S or S-M), class 4 (S-S), class 5 (M-M), and class 6 (U-U). Soft-clipped reads in classes 2, 3 and 5 are used to find breakpoints. The read pairs in classes 1 and 2 are considered as one-end unmapped reads, which are anchored at the upstream or downstream of the breakpoints. These one-end unmapped reads are used to annotate MGEs inserted. **c** Clipped subsequences (in yellow) at the breakpoints are used to check the integrity of the reads aligned. Breakpoints that are poorly aligned with clipped subsequences are discarded. **d** The vicinity of the breakpoint is estimated if a few nucleotides are the same. Such situation could occur when the inserted fragment has target site duplication. Read 1 at the beginning of MGEs might be the same as those in the downstream of the breakpoints. Alternatively, read 2 at the end of MGEs might be the same as those in upstream

By using the reads in these categories, the paired-end reads are further classified and retrieved (see Fig. 1a and b): (i) one-end unmapped (i.e. paired-end reads corresponding to M-U or U-M); (ii) soft-clipped with an unmapped read at one end (i.e. S-U or U-S); (iii) soft-clipped with a mapped read at one end (i.e. S-M or M-S); (iv) both soft-clipped (i.e. S-S); (v) both mapped (i.e. M-M); (vi) both unmapped (i.e. U-U). If the distance between the read pairs belonging to the class (iii), (iv) or (v) is significantly longer than the average insert size (default > 500 bp), the read that has higher mapping quality is treated as the anchor so that the mate read is manipulated as unmapped.

### Prediction of breakpoints

**Fig. 2:**
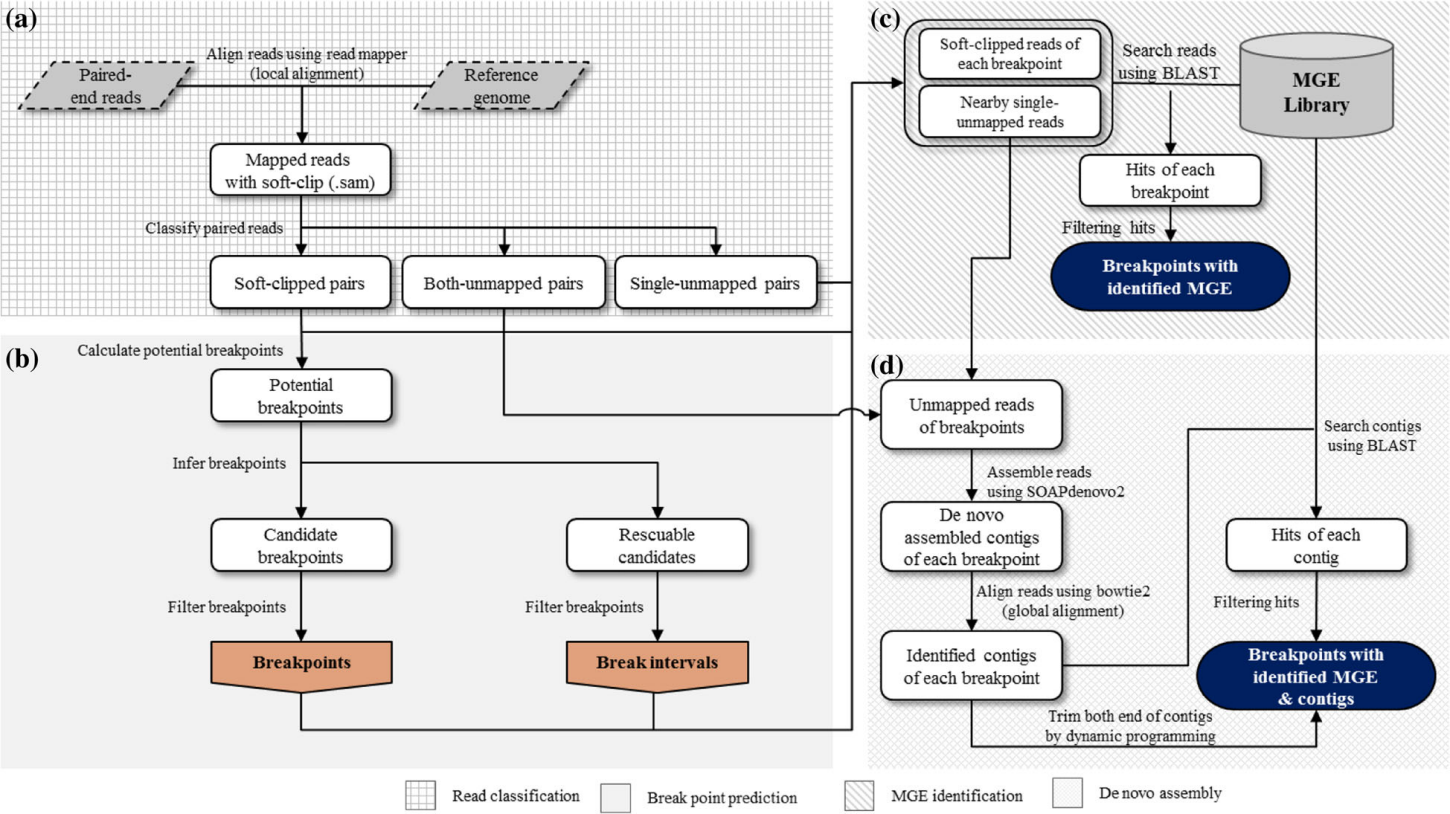
Overview of the iMGEins pipeline. iMGEins has four phases: **a** read classification, **b** breakpoint prediction, **c** MGE identification, and **d** de novo assembly

Candidate breakpoints are predicted by taking five consecutive steps shown in Fig. 2b. First, the initial breakpoints are estimated by taking into account the three aspects: (i) the length of unaligned substrings in the soft-clipped reads (default > 5 bp); (ii) the number of supporting reads for breakpoints (default > 10% of the coverage); (iii) average base quality of clipped bases (default > 59 phred score). The supporting reads for the breakpoints can be either upstream-support (towards the 5′-end) or downstream-support (towards the 3′-end). It is desirable to have both upstream- and downstream-support except a few cases such as 5′ truncated insertions. When the initial breakpoints have a sufficient number of upstream- or downstream-support reads, the breakpoints are retained as candidate breakpoints.

Second, we adapt profile analysis from multiple sequence alignments to filter out false positive mappings (Fig. 1c). The unaligned sequences of soft-clipped reads at each breakpoint are aligned, and sufficiently long regions (default > 10 bp) that are next to the breakpoints are profiled. If the similarity among sequences is low, the breakpoints are discarded (default > 95%). In this step, the number of A or T bases in soft-clipped region are also considered since PolyA signal is an important feature of retrotransposition for LINE and Alu [45].

Third, breakpoints that overlap with the sufficient reads are eliminated from the pool of candidates to discard false positive breakpoints. Some reads could be soft-clipped by sequencing errors and/or low-quality bases at the end of the reads. We set 10% for the ratio of soft-clipped reads at the breakpoints to call haplotype insertions, and 90% otherwise [46].

Fourth, the vicinity of the breakpoints (i.e. a short interval between breakpoints that is caused by some events such as target site duplication) is estimated. A few nucleotides at the beginning of the inserted fragments could be the same as those in the downstream of the breakpoints (Fig. 1d). Such results might be ascribed to a target site duplication (TSD) or a random event. In order to deal with such situation, iMGEins allows a short interval for the breakpoints as the vicinity of breakpoints (default < 25 bp).

Lastly, false breakpoints caused by short indels, long deletions, or tandem repeats are filtered out. If tandem repeats exist, reads are mapped with soft-clipping that allows iMGEins to detect as breakpoints of MGE insertion. Therefore, we eliminate such breakpoints by checking short indels nearby breakpoints, read depth of both sides of breakpoints, and whether the soft-clipped sequence is identical to the reference sequences of breakpoints.

### Identification of MGEs using one-end unmapped reads and soft-clipped reads

The breakpoints that are obtained in the previous step are further analyzed to annotate MGEs. One-end unmapped or soft-clipped reads on the upstream or downstream of the candidate breakpoints are searched (Fig. 2c). If one end is properly mapped, the other end is not mapped or discordantly mapped on the reference genome. The range should be selected by considering the insert size of the sequencing reads library. From the candidate discordant read pairs (S-U, U-S, M-U or U-M) of each breakpoint, the one-end unmapped reads (U) and soft-clipped reads (S) of each breakpoint are searched against the MGE sequence library such as Repbase [47] to find the homology with known MGEs. The results of BLAST [48] search are filtered by user-defined thresholds (default setting: similarity > 90%; hit length > 70% of the average read length). After performing majority voting, only the most confident MGE for each breakpoint is reported. The results are saved in the GFF format, which could be used for further analysis.

### Identification of MGEs using de novo assembly

In the step described above, iMGEins reports the most similar known MGEs for the inserted sequences at the breakpoints. However, novel insertions could also be certain types of variants or novel sequences. Furthermore, MGEs could not be matched correctly if unmapped reads nearby the breakpoints are very short or partially matched to remotely homologous MGEs.

**Fig. 3:**
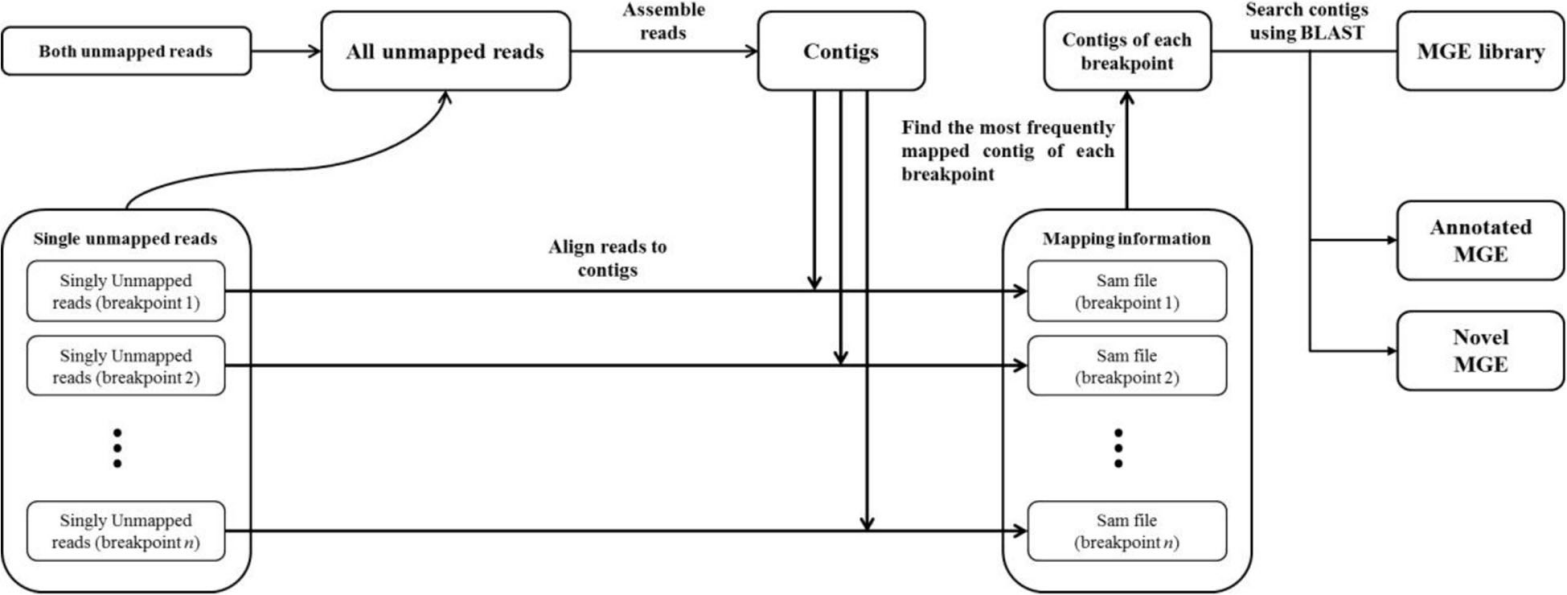
Assembly of inserted MGEs. Unmapped paired reads and one-end unmapped reads are collected in the read classification step and MGE identification step. Reads are assembled to contigs, which include the fragments inserted into each breakpoint. One-end unmapped reads are used to find the corresponding contigs inserted to a specific breakpoint. The contigs are searched against the MGE library for annotation

In order to find and annotate novel MGEs at each breakpoint, we collected and assembled reads that are unmapped and one-end mapped (Figs. 2d and 3). First, both unmapped paired-reads are collected in the read classification step. All one-end unmapped reads nearby each breakpoint are collected in this step. For a more precise assembly, we further added soft-clipped reads that support the breakpoint. These unmapped and soft-clipped reads are assembled using SOAPdenovo2 [49] with the k-mer size of 51. Second, contigs shorter than the sufficient length (default value < 500) are filtered out since our main purpose is to find long insertions. Third, one-end unmapped reads of each breakpoint are aligned to the assembled contigs using Bowtie2 with a sensitive preset and at most five distinct alignments for each read (−k = 5). Fourth, the most probable MGE for each breakpoint is annotated after performing majority voting. In this step, contigs with a small number of reads mapped (default < 2) are filtered out.

Finally, we searched the contigs against the MGE library to find whether the identified contig is homologous to the known MGEs (Figs. 2d and 3). We consider a contig to be novel if it is not identified by BLAST search with the annotated MGE library. At the same time, the most proper contig is trimmed out by aligning the soft-clipped sequence using dynamic programming (local alignment with gap open = − 5; gap extension = − 2; substitution = − 3; match = 1). If the optimal score of dynamic programming is lower than 75% of the length of the longest clipped sequence, we do not report the sequence to avoid false positives. This procedure is also performed with reverse-complement sequence, but reports only one strand result which aligns more properly. By these procedures, iMGEins is able to find the inserted sequences successfully and their boundaries more precisely.

### Performance evaluation

In order to evaluate the performance of iMGEins, the recall and precision rates were measured separately for the breakpoints and the predicted MGE types. Since some programs do not predict the MGE types, it would be fair to compare the accuracy for each category separately. For the breakpoints, we consider the recall and precision rates. The recall rate is measured as the ratio of correctly predicted breakpoints to all inserted MGEs in each simulated genome. The precision rate is measured as the ratio of correctly predicted breakpoints to all breakpoints predicted by each program. The breakpoints are considered to be correctly predicted if they are within the 20 bps upstream or downstream of the real insertion. Some programs predict breakpoints based on the discordant reads pair mapping information, and thus their prediction is not precise. We thus allowed approximated boundary for the true positive hits. A detailed comparison is provided in the Results section.

For the accuracy of the MGE prediction, we consider the recall and precision rates. In the case of iMGEins, two different MGE predictions are made from one-end unmapped reads and assembled contigs. We consider the prediction as false positive if iMGEins finds more than one MGE type. We applied rather stringent measures in order to evaluate the performance of iMGEins more precisely. For the novel MGEs, we consider the prediction as true positive if iMGEins reports accurate insertion sequences. Since the information of MGE types is unavailable, the accuracy is determined by whether the contigs are assembled correctly or not.

### Computational complexity of iMGEins

In processing the data with iMGEins, breakpoints are predicted based on the soft-clipped reads. The inserted MGEs are identified by one-end unmapped reads or read assembly. The computational complexity of read classification step is O(*n*), where *n* is the total number of sequencing reads. To obtain putative breakpoints, all soft-clipped reads should be investigated. Since all soft-clipped reads are iterated, the amount of time required is O(*s*), where *s* is the number of soft-clipped reads. For example, NA12878 data contains about 500 million soft-clipped reads, which correspond to more than 20% of the entire reads. Subsequently, iMGEins finds accurate breakpoints by filtering putative breakpoints. The amount of time required for this step is O(*p*), where *p* is the number of putative breakpoints.

Finally, the inserted MGE types are identified by searching one-end unmapped reads and soft-clipped reads nearby breakpoints. For long DNA fragment size and high coverage sequencing data sets, iMGEins shows the highest memory usage and computational time in all processes. Therefore, the computational complexity of this step is O(u*f), where *u* is the number of one-end unmapped reads nearby breakpoints, and *f* is the fragment size of the data set. A BLAST search is also performed, but it spends relatively little time, compared to the entire processing time. Specifically, BLAST search took 78 mins for NA12878 data, compared to 1606 mins for the entire processing time.

In the assembly of MGEs, all unmapped reads in each data set are assembled by SOAPdenovo2, and one-end unmapped reads are aligned to contigs by bowtie2. After that, the most relevant contigs of each breakpoint are searched against the MGE database. For such process, the computational complexity is dependent on the coverage of the data set, which is O(m*(*C*_1_ + *C*_2_)). Here, *m* is the length of the most relevant contig; *C*_1_ and *C*_2_ are the length of the longest soft-clipped sequences of the 3′-end and 5′-end, respectively.

We measured the running time of iMGEins for the simulated data sets with different coverage. The number of breakpoints is almost the same for each simulation data set. It took about 9 mins and 28 mins for the 30x and the 90x coverage data, respectively. This result shows that the running time of iMGEins linearly increases with respect to the read coverage. We also measured the running time for each step of iMGEins using the large-scale data set, NA12878. The size of the data is > 1.6 TB, and it has more than three thousand breakpoints. The entire process took about a day on Intel(R) Xeon(R) CPU E5–2699 v4 @ 2.20GHz with 32 cores. Specifically, breakpoint prediction and de novo assembly took 52% of the processing time. It is apparent that the time complexity of the two steps is tightly correlated with the number of breakpoints and the number of one-end unmapped reads.

## Results

We evaluated iMGEins by generating genomes that contain simulated MGEs and novel insertions. The performance of iMGEins was compared with current state-of-the-art programs, RetroSeq [13], TEMP [31], PoPoolationTE [38], and MELT [39]. For each type of MGEs, the recall and precision rates were compared. In addition, we tested iMGEins by using real sequencing reads obtained from an individual human genome (NA12878). The performance was measured in terms of the recall and precision rates. The overall accuracy was calculated by averaging the recall and precision rates.

### Evaluation of iMGEins on the simulated sequencing data

In order to evaluate the performance of iMGEins, we first generated sequencing reads from the human genomes with the simulated MGEs of different types and the SNV ratio. In particular, the first set of simulated genomes contain 1000 MGE insertions with LINE, SINE, LTR, and DNA transposon. A total of 500 MGE sequences were generated with or without SNV. Specifically, 200 MGEs were generated without SNV, while 300 MGEs with 10–50% SNVs. In addition, 500 random sequences with similar length to the MGEs were generated as control (Additional file 1: Table S1). The second set of simulated genomes contain 80 known MGEs of primate species, 80 known MGEs of human, and 80 novel sequences (Additional file 1: Table S2). It should be noted that novel insertion sequences were used to test the ability of detecting novel MGE insertions. In order to evaluate the performance of different coverage, high (90x) and low (30x) coverage of sequencing reads were generated from the second genomes.

The MGE fragments were inserted at random positions, but avoiding ‘N’ masking regions. All of the inserted fragments are longer than 500 bps. The inserted MGEs contain only A, C, G and T, and do not include any ambiguous nucleotides. This is because RetroSeq [13] allows only A, C, G, and T. In order to measure the accuracy of finding MGEs with target site duplication (TSD), simulated MGEs in the second simulation data set have random TSDs of 2–9 bps. From the simulated genomes described above, paired sequencing reads were obtained with the following parameters: read length = 100; mean coverage = 30 and 90; mean insert length = 400; standard deviation of insert length = 20. The error model was chosen for the default built-in quality score profile of HiSeq2000 with an empirical error model (~ 0.8% error rate). These simulated sequencing reads were aligned against the human reference genome (hg19) by using the read mapping program BWA [43], as suggested in each of the MGE finding programs TEMP [31] and RetroSeq [13]. The read mapping step is required for most of the MGE finding programs with default options.

As shown in Fig. 1d, iMGEins allows a short interval (at most 25 bp) for breakpoints to account for TSD or random events. Other programs also report such intervals. TEMP [31] reports breakpoint intervals at the longest fragment insert length because it identifies breakpoints by using discordantly mapped reads first. RestroSeq [13] reports breakpoints with 1 bp interval only, PoPoolationTE [38] reports breakpoint intervals at the longest read length and MELT [39] reports within 25 bp. Therefore, we considered that the identified breakpoints are true positives if the predicted position is within 20 bps upstream or downstream of the actual position.

The performance of iMGEins was compared with the state-of-the-art MGE discovery programs, TEMP [31], RetroSeq [13], PoPoolationTE [38], and MELT [39]. Notably, iMGEins, TEMP, and MELT found most of the breakpoints of MGEs without SNVs in the evaluation with the first simulation data (Table 1). The average recall rate of iMGEins was 97%, while TEMP was 96% and MELT was 98.5%. For the MGEs with 10% of SNVs, iMGEins outperformed the four other methods in terms of the recall rate (95% for iMGEins vs. 0% for TEMP, 23% for RetroSeq, 47% for PoPoolationTE, and 35% for MELT). For the four other programs, the recall rates drop significantly as the ratio of SNVs increases. The average precision rate for iMGEins is 97.89%, while TEMP is 89.72% and MELT is 96.04 (Table 1). In particular, iMGEins predicts 21 false positives, while TEMP predicts 22, PoPoolationTE predicts 16,109, RetroSeq predicts 663, and MELT predicts 9.

**Table 1:** Recall rates of breakpoint detection of iMGEins and novel insertions, popoolationTE, TEMP, RetroSeq and MELT in the simulation set 1

Simulation type	MGE type	Ratio of SNVs	iMGEins	PoPoolationTE	TEMP	RetroSeq	MELT
Non variant (%)	LINE		100.00	22.00	90.00	2.00	98.00
	SINE		94.00	84.00	96.00	24.00	98.00
	LTR		96.00	86.00	100.00	62.00	100.00
	DNA		98.00	86.00	98.00	26.00	98.00
SNV (%)	LINE	10%	100.00	10.00	0.00	0.00	40.00
		20%	100.00	0.00	0.00	20.00	0.00
		30%	100.00	0.00	0.00	0.00	0.00
		40%	100.00	0.00	0.00	10.00	0.00
		50%	90.00	0.00	0.00	25.00	0.00
	SINE	10%	95.00	60.00	0.00	25.00	10.00
		20%	100.00	0.00	0.00	40.00	0.00
		30%	100.00	0.00	0.00	25.00	0.00
		40%	95.00	5.00	0.00	35.00	0.00
		50%	100.00	0.00	0.00	50.00	0.00
	LTR	10%	90.00	70.00	0.00	45.00	55.00
		20%	100.00	0.00	0.00	55.00	0.00
		30%	85.00	0.00	0.00	50.00	0.00
		40%	95.00	0.00	0.00	40.00	0.00
		50%	95.00	0.00	0.00	60.00	0.00
Random (%)	LINE		98.00	3.33	0.00	34.67	0.00
	SINE		98.67	6.00	0.00	36.00	0.00
	LTR		98.00	0.67	0.00	34.00	0.00
	DNA		100.00	0.00	0.00	36.00	0.00
True Positives			975	183	192	328	218
False Positives			21	16,109	22	663	9

**Fig. 4:**
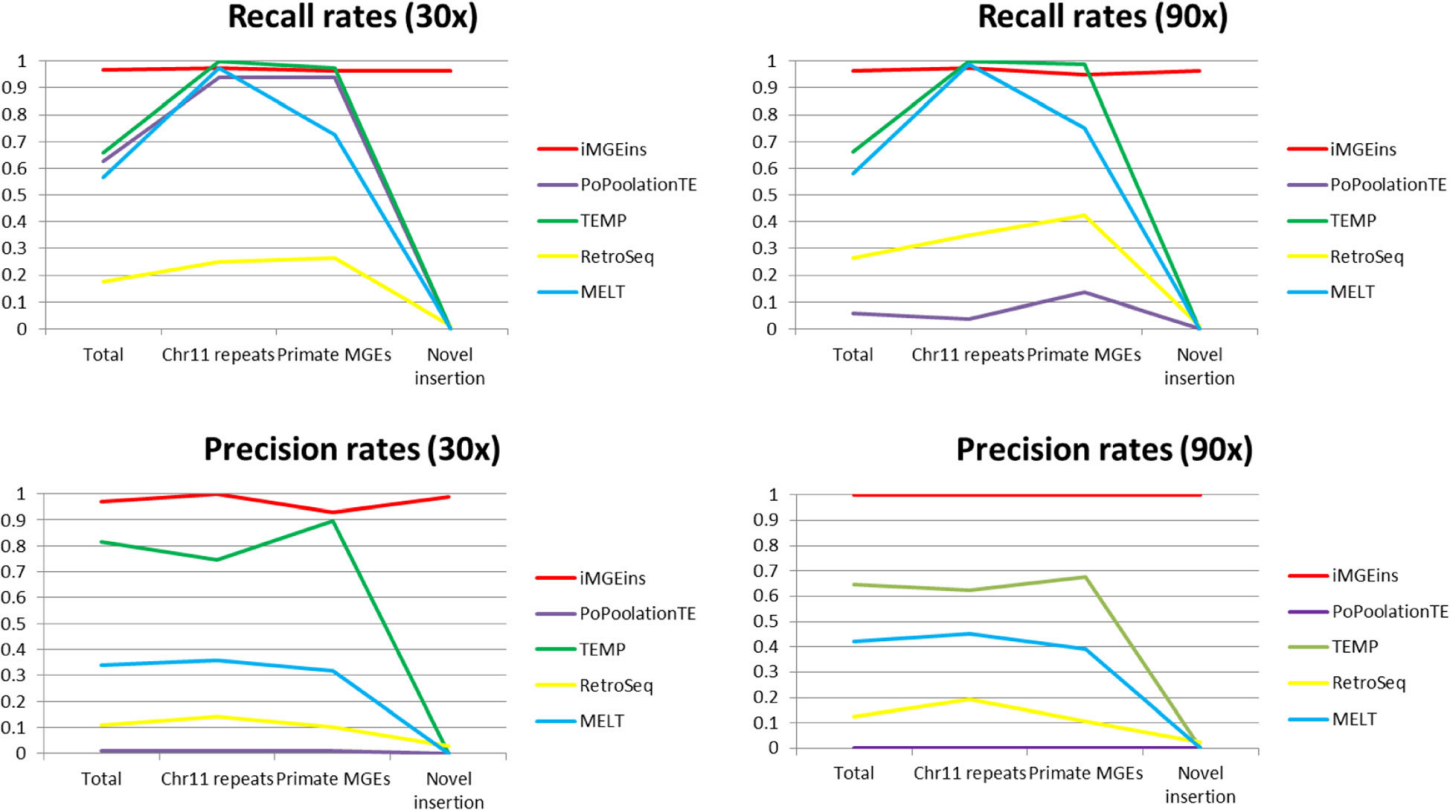
Recall and precision rates of breakpoints identified for the second simulated data (Additional file 1: Table S2) by iMGEins, PoPoolationTE, TEMP, RetroSeq, and MELT

In the evaluation with the second simulation data, two sets of different coverage were used to evaluate the five programs. Overall, iMGEins outperformed other methods in finding breakpoints (Fig. 4). iMGEins located most of the breakpoints with few false positives (97.07 and 100% precision rate) in both low and high coverage. Notably, iMGEins correctly identified novel insertion sequences (96.25%). In contrast, other programs showed much lower recall and precision rates for novel MGEs. TEMP showed comparable performance in finding known MGEs (on average, 96.88% recall rate by iMGEs vs 98.75% by TEMP with 30x coverage; 96.25% recall rate by iMGEs vs 99.38% by TEMP with 90x coverage), but could not find novel MGEs (96.25% recall rate by iMGEs vs 0% by TEMP with 30x coverage; 96.25% recall rate by iMGEs vs 0% by TEMP with 90x coverage). MELT showed good performance only for the known MGEs, not for the divergent MGEs (Fig. 4). In terms of the precision rates, iMGEins also showed significantly better performance (97.07% by iMGES, 81.44% by TEMP and 10.99% by RetroSeq with 30x coverage; 100% by iMGES, 64.63% by TEMP and 12.43% by RetroSeq with 90x coverage).

In addition to finding the breakpoints of MGEs, it is also important to annotate the inserted MGE sequences. For this, iMGEins assembles the MGE contigs, and predicts MGE types as well. As of now, a few programs can predict MGE types by assembling MGEs, but only to a limited degree. We compared the performance of predicting the types of inserted MGEs in the second simulation data set (Table 2). iMGEins achieved very high recall rates (96.67 and 95.83% for 30x and 90x, respectively) and the best precision rate (100% for 30x and 90x) in predicting MGE types. Since TEMP reports multiple distinct MGEs on the same breakpoint, there are differences between the number of breakpoints and the number of identified MGEs. Although TEMP showed 98.75 and 99.38% recall rate for 30x and 90x, respectively, it only found known MGEs. RetroSeq reported with 10.99% precision rate, which is the lowest among the three programs.

**Table 2:** Performance of MGE detection in iMGEins, popoolationTE, TEMP, RetroSeq and MELT in simulation set 2

	Low coverage (30x)					Hogh coverage (90x)				
	iMGEins	PoPoolationTE	TEMP	RetroSeq	MELT	iMGEins	PoPoolationTE	TEMP	RetroSeq	MELT
True positive										
Human MGEs	78	75	80	19	72	75	2	80	27	74
Primate MGEs	77	74	78	20	37	78	10	79	31	39
Novel insertions	77	0	0	1	0	77	0	0	0	0
Total	232	149	158	40	109	231	12	159	58	113
False Positive										
Human MGEs	0	0	31	121	6	0	1	56	117	5
Primate MGEs	0	1	10	188	21	0	1	40	289	21
Novel insertions	0	0	0	33	0	0	0	0	43	0
Total	0	1	41	342	27	0	2	96	449	26
Recall (%)										
Human MGEs	97.50	93.75	100.00	23.75	90.00	93.75	2.50	100.00	33.75	92.50
Primate MGEs	96.25	92.50	97.50	25.00	46.25	97.50	12.50	98.75	38.75	48.75
Novel insertions	96.25	-^a^	–	1.25	–	96.25	–	–	0.00	–
Average										
Complete^b^ MGE only^c^	96.67	62.08 93.13	65.83 98.75	16.67	45.42 68.13	95.83	5.00 7.5	66.25 99.38	24.17	47.08 70.63
Precision (%)										
Human MGEs	100.00	100.00	72.07	13.57	92.31	100.00	66.67	58.82	18.75	93.67
Primate MGEs	100.00	98.67	88.64	9.62	63.79	100.00	90.91	66.39	9.69	65.00
Novel insertions	100.00	–	–	2.94	–	100.00	–	–	0.00	–
Average	100.00	99.333	79.40	10.47	80.15	100.00	85.71	62.35	11.44	81.29

^a^These programs cannot find novel insertions

^b^The rates for the entire test case

^c^The rates without the novel insertion category

Since iMGEins was designed to report MGEs inserted, we assessed how completely the MGEs are assembled and annotated. As a stringent measure to decide the MGEs as true positive, we allowed only two base differences in both ends of the fragments. iMGEins successfully found novel MGEs that are not in the database. When iMGEins locates the breakpoints, it perfectly predicts the MGE sequences for the sequences inserted. Among the programs compared, only RetroSeq algorithm provides a procedure for predicting novel MGEs.

### Evaluation on the real human genomes

In order to test the performance of iMGEins on real sequencing data, we used a well-known high coverage (> 85x) Illumina HiSeq dataset from the 1000 Genome Project (NA12878). Since this dataset has PacBio long reads, it has been effectively used for non-reference TE detection. A BAM file mapped by BWA [43] was obtained from ftp://ftp.1000genomes.ebi.ac.uk/vol1/ftp/technical/working/20120117_ceu_trio_b37_decoy/. A validated set of insertions was obtained for comparison from the 1000 Genome Pilot Project (ftp://ftp.ncbi.nlm.nih.gov/pub/dbVar/data/Homo_sapiens/by_study/vcf/) [50]. The coordinates of breakpoints were converted from hg18 to hg 19 using liftOver utility. The results of iMGEins was compared with those of TEMP [31], RetroSeq [13], Tea [22], Tangram [14] and MELT [39]. The running results of RetroSeq [13], Tea [22] and Tangram [14] were downloaded from ftp://ftp-mouse.sanger.ac.uk/other/tk2/RetroSeq/CEU_trio/. Consensus sequences for ALU and L1 elements were obtained from RetroSeq [13].

In the evaluation, iMGEins reported 3811 breakpoints that are annotated as ALU or L1 insertions with stringent options (soft-clip maps > 10). For TEMP [31], post-processing was performed as suggested by the authors [31]. Insertions were filtered when they are supported by less than 20 reads and have an allele frequency of less than 20% for the high coverage data. The numbers of breakpoints identified by each program are summarized in Table 3. iMGEins successfully identified the positions of breakpoints at the base pair resolution. When we allow 200 bp proximity around the real breakpoint for correct prediction, the performance of the programs are comparable. However, as the resolution decreases to 20 bp, iMGEins shows the highest recall rates. Notably, iMGEins accurately predicted over 90% of breakpoints within 20 bp of the annotated breakpoints. Several breakpoints outside of the 20 bps proximity are due to TSD. Although TEMP [31] also showed a comparable recall rate at 20 bp resolution, the coordinates of breakpoints reported by TEMP [31] span excessively large ranges (on average 293.40 bp), compared to iMGEins that only allows 25 bp intervals (on average 4.52 bp). The fact that the breakpoints are more accurately predicted is one of the advanced features of iMGEins. Overall, 68.59, 87.65, 42.25, 21.18, and 91.5% of the breakpoints predicted within 100 bps proximity of the real breakpoints retained for the threshold of 20 bp proximity by Tangram [14], Tea [22], RetroSeq [13], MELT [39], and iMGEins, respectively.

**Table 3:** Comparison of breakpoint prediction for the NA12878 dataset for iMGEins, Tangram, Tea, TEMP, RetroSeq and MELT

Proximity around breakpoints^a^	iMGEins	Tangram	Tea	TEMP	RetroSeq	MELT
Breakpoints ±100	400	433	397	394	426	439
Breakpoints ±20	397	421	393	394	305	421
Breakpoints ±10	366	297	348	363	180	93

^a^The distance around the annotated breakpoints, which is allowed to be consider as true positive

Notably, iMGEins could successfully reports additional breakpoints that are not included in the experimental evaluation set that we used in the comparison. After manual investigation using IGV carefully, we made an observation that the additional breakpoint might be the real breakpoints (Additional file 1: Figure S2).

### Genome misassembly rectification and PCR validation

In addition to predicting novel MGEs with high precision rates, iMGEins can also locate the chimeric points in assembled genomes. In order to evaluate its capability to correct mis-assembly, we applied iMGEins to the assembly of the minke whale genome [51], and validated the results by using PCR. By re-aligning the whole genome sequencing data to the genome sequence and applying iMGEins, we could identify 765 chimeric points with a significant number of supporting reads. Among these, iMGEins assembled the inserted sequences for 9 breakpoints with a sufficient number of aligned reads to long contig. For PCR validation, we randomly selected three points that contain the assembly of inserted sequences. Results from PCR were compared with the inserted sequences predicted from iMGEins to find that the homology is about 96% (Additional file 1: Figures S3–S4 and Tables S3–S5).

## Conclusion

MGEs play important roles as a driver of genomic instability. Individual human genomes have shown recent insertions of MGEs that are related with phenotypic changes such as cancer. In order to study the dynamics of MGEs in individual genomes, iMGEins was developed to accurately detect breakpoints and report inserted MGEs. Compared with other programs, iMGEins has valuable features of identifying novel MGEs and assembling the MGEs inserted. In addition, iMGEins can find genome mis-assembly, which was validated by experimental studies on the minke whale genome.

## Availability and requirements

Project name: iMGEins

Project home page: https://github.com/DMnBI/iMGEins

Operation system: Linux

Programming language: Java

Other requirements: Java version 8 or higher

License: GNU GPL

Any restrictions to use by non-academics: licence needed

## Additional file

Additional file 1: A PDF file with Tables S1–S5 and Figures S1–S3. (PDF 454 kb)

## Abbreviations

LTR: Long terminal repeat
MGE: Mobile genetic element
SAM: Sequence alignment map
SNPs: Single nucleotide polymorphisms
TSD: Target site duplication

## References

[1] Hide G, Tilley A (2001). Use of mobile genetic elements as tools for molecular epidemiology. Int J Parasitol.

[2] Arkhipova IR (2005). Mobile genetic elements and sexual reproduction. Cytogenet Genome Res.

[3] Coyne MJ, Roelofs KG, Comstock LE (2016). Type VI secretion systems of human gut Bacteroidales segregate into three genetic architectures, two of which are contained on mobile genetic elements. BMC Genomics.

[4] Georgiev GP (1984). Mobile genetic elements in animal cells and their biological significance. Eur J Biochem.

[5] Makarova KS, Wolf YI, van der Oost J, Koonin EV (2009). Prokaryotic homologs of Argonaute proteins are predicted to function as key components of a novel system of defense against mobile genetic elements. Biol Direct.

[6] Miller WJ, Capy P (2004). Mobile genetic elements as natural tools for genome evolution. Methods Mol Biol.

[7] Miller WJ, Capy P (2006). Applying mobile genetic elements for genome analysis and evolution. Mol Biotechnol.

[8] Terry RS, Smith JE, Duncanson P, Hide G (2001). MGE-PCR: a novel approach to the analysis of toxoplasma gondii strain differentiation using mobile genetic elements. Int J Parasitol.

[9] Chen K, Wallis JW, McLellan MD, Larson DE, Kalicki JM, Pohl CS, McGrath SD, Wendl MC, Zhang Q, Locke DP (2009). BreakDancer: an algorithm for high-resolution mapping of genomic structural variation. Nat Methods.

[10] Kai Y, Schulz MH, Long Q, Apweiler R, Ning Z (2009). Pindel: a pattern growth approach to detect break points of large deletions and medium sized insertions from paired-end short reads. Bioinformatics.

[11] Rishishwar L, Marino-Ramirez L, Jordan IK. Benchmarking computational tools for polymorphic transposable element detection. Brief Bioinform. 2016;18.6:908-18. 10.1093/bib/bbw072 PMC5808724 27524380

[12] David M, Mustafa H, Brudno M (2013). Detecting Alu insertions from high-throughput sequencing data. Nucleic Acids Res.

[13] Keane TM, Wong K, Adams DJ (2013). RetroSeq: transposable element discovery from next-generation sequencing data. Bioinformatics.

[14] Wu J, Lee WP, Ward A, Walker JA, Konkel MK, Batzer MA, Marth GT (2014). Tangram: a comprehensive toolbox for mobile element insertion detection. BMC Genomics.

[15] Ewing AD (2015). Transposable element detection from whole genome sequence data. Mob DNA.

[16] Kroon M, Lameijer EW, Lakenberg N, Hehir-Kwa JY, Thung DT, Slagboom PE, Kok JN, Ye K (2016). Detecting dispersed duplications in high-throughput sequencing data using a database-free approach. Bioinformatics.

[17] Quinlan AR, Clark RA, Sokolova S, Leibowitz ML, Zhang Y, Hurles ME, Mell JC, Hall IM (2010). Genome-wide mapping and assembly of structural variant breakpoints in the mouse genome. Genome Res.

[18] Xiong W, He L, Li Y, Dooner HK, Du C (2013). InsertionMapper: a pipeline tool for the identification of targeted sequences from multidimensional high throughput sequencing data. BMC Genomics.

[19] Hawkey J, Hamidian M, Wick RR, Edwards DJ, Billman-Jacobe H, Hall RM, Holt KE (2015). ISMapper: identifying transposase insertion sites in bacterial genomes from short read sequence data. BMC Genomics.

[20] Jiang C, Chen C, Huang Z, Liu R, Verdier J (2015). ITIS, a bioinformatics tool for accurate identification of transposon insertion sites using next-generation sequencing data. BMC Bioinformatics.

[21] Henaff E, Zapata L, Casacuberta JM, Ossowski S (2015). Jitterbug: somatic and germline transposon insertion detection at single-nucleotide resolution. BMC Genomics.

[22] Lee E, Iskow R, Yang L, Gokcumen O, Haseley P, Luquette LJ, Lohr JG, Harris CC, Ding L, Wilson RK (2012). Landscape of somatic retrotransposition in human cancers. Science.

[23] Mohiyuddin M, Mu JC, Li J, Bani Asadi N, Gerstein MB, Abyzov A, Wong WH, Lam HY (2015). MetaSV: an accurate and integrative structural-variant caller for next generation sequencing. Bioinformatics.

[24] Thung DT, de Ligt J, Vissers LE, Steehouwer M, Kroon M, de Vries P, Slagboom EP, Ye K, Veltman JA, Hehir-Kwa JY (2014). Mobster: accurate detection of mobile element insertions in next generation sequencing data. Genome Biol.

[25] Tempel S, Pollet N, Tahi F (2012). ncRNAclassifier: a tool for detection and classification of transposable element sequences in RNA hairpins. BMC Bioinformatics.

[26] Tica J, Lee E, Untergasser A, Meiers S, Garfield DA, Gokcumen O, Furlong EE, Park PJ, Stutz AM, Korbel JO (2016). Next-generation sequencing-based detection of germline L1-mediated transductions. BMC Genomics.

[27] Hormozdiari F, Hajirasouliha I, Dao P, Hach F, Yorukoglu D, Alkan C, Eichler EE, Sahinalp SC (2010). Next-generation VariationHunter: combinatorial algorithms for transposon insertion discovery. Bioinformatics.

[28] Kang H, Zhu D, Lin R, Opiyo SO, Jiang N, Shiu SH, Wang GL (2016). A novel method for identifying polymorphic transposable elements via scanning of high-throughput short reads. DNA Res.

[29] Helman E, Lawrence MS, Stewart C, Sougnez C, Getz G, Meyerson M (2014). Somatic retrotransposition in human cancer revealed by whole-genome and exome sequencing. Genome Res.

[30] Platzer A, Nizhynska V, Long Q (2012). TE-locate: a tool to locate and group transposable element occurrences using paired-end next-generation sequencing data. Biology (Basel).

[31] Zhuang J, Wang J, Theurkauf W, Weng Z (2014). TEMP: a computational method for analyzing transposable element polymorphism in populations. Nucleic Acids Res.

[32] Gilly A, Etcheverry M, Madoui MA, Guy J, Quadrana L, Alberti A, Martin A, Heitkam T, Engelen S, Labadie K (2014). TE-Tracker: systematic identification of transposition events through whole-genome resequencing. BMC Bioinformatics.

[33] Chen K, Chen L, Fan X, Wallis J, Ding L, Weinstock G (2014). TIGRA: a targeted iterative graph routing assembler for breakpoint assembly. Genome Res.

[34] Fiston-Lavier AS, Barron MG, Petrov DA, Gonzalez J (2015). T-lex2: genotyping, frequency estimation and re-annotation of transposable elements using single or pooled next-generation sequencing data. Nucleic Acids Res.

[35] Nakagome M, Solovieva E, Takahashi A, Yasue H, Hirochika H, Miyao A (2014). Transposon insertion finder (TIF): a novel program for detection of de novo transpositions of transposable elements. BMC Bioinformatics.

[36] Robb SM, Lu L, Valencia E, Burnette JM, Okumoto Y, Wessler SR, Stajich JE (2013). The use of RelocaTE and unassembled short reads to produce high-resolution snapshots of transposable element generated diversity in rice. G3 (Bethesda).

[37] Linheiro RS, Bergman CM (2012). Whole genome resequencing reveals natural target site preferences of transposable elements in Drosophila melanogaster. PLoS One.

[38] Kofler R, Betancourt AJ, Schlotterer C (2012). Sequencing of pooled DNA samples (Pool-Seq) uncovers complex dynamics of transposable element insertions in Drosophila melanogaster. PLoS Genet.

[39] Gardner EJ, Lam VK, Harris DN, Chuang NT, Scott EC, Pittard WS, Mills RE, Devine SE, Genomes Project, C (2017). The Mobile Element Locator Tool (MELT): population-scale mobile element discovery and biology. Genome Res.

[40] Tubio JM, Li Y, Ju YS, Martincorena I, Cooke SL, Tojo M, Gundem G, Pipinikas CP, Zamora J, Raine K (2014). Mobile DNA in cancer. Extensive transduction of nonrepetitive DNA mediated by L1 retrotransposition in cancer genomes. Science.

[41] Nelson MG, Linheiro RS, Bergman CM (2017). McClintock: an integrated pipeline for detecting transposable element insertions in whole-genome shotgun sequencing data. G3 (Bethesda).

[42] Langmead B, Salzberg SL (2012). Fast gapped-read alignment with bowtie 2. Nat Methods.

[43] Li H, Durbin R (2009). Fast and accurate short read alignment with burrows–wheeler transform. Bioinformatics.

[44] Lee WP, Stromberg MP, Ward A, Stewart C, Garrison EP, Marth GT (2014). MOSAIK: a hash-based algorithm for accurate next-generation sequencing short-read mapping. PLoS One.

[45] Dewannieux M, Heidmann T (2005). Role of poly(A) tail length in Alu retrotransposition. Genomics.

[46] Shen Y, Wan Z, Coarfa C, Drabek R, Chen L, Ostrowski EA, Liu Y, Weinstock GM, Wheeler DA, Gibbs RA (2010). A SNP discovery method to assess variant allele probability from next-generation resequencing data. Genome Res.

[47] Bao W, Kojima KK, Kohany O (2015). Repbase update, a database of repetitive elements in eukaryotic genomes. Mob DNA.

[48] McGinnis S, Madden TL (2004). BLAST: at the core of a powerful and diverse set of sequence analysis tools. Nucleic Acids Res.

[49] Luo R, Liu B, Xie Y, Li Z, Huang W, Yuan J, He G, Chen Y, Pan Q, Liu Y (2012). SOAPdenovo2: an empirically improved memory-efficient short-read de novo assembler. Gigascience.

[50] Consortium TGP (2010). A map of human genome variation from population-scale sequencing. Nature.

[51] Yim HS, Cho YS, Guang X, Kang SG, Jeong JY, Cha SS, Oh HM, Lee JH, Yang EC, Kwon KK (2014). Minke whale genome and aquatic adaptation in cetaceans. Nat Genet.

