# Supplementary material for: iMGEins: detecting novel mobile genetic elements inserted in individual genomes
Source: BMC Genomics. 2018 Dec 18;19:944. doi: 10.1186/s12864-018-5290-9 (PMC6299635; doi:10.1186/s12864-018-5290-9)
Supplement: Supplementary file 1 — A PDF file with Tables S1–S5 and Figures S1–S3. (PDF 454 kb) [file 12864_2018_5290_MOESM1_ESM.pdf]

Supplementary Material

Table S1. Summary of the simulated genomes with different MGE types.

	SNV rates						Random sequences[1]
	0%	10%	20%	30%	40%	50%	
LINE	50	20	20	20	20	20	150
SINE	50	20	20	20	20	20	150
LTR retrotransposons	50	20	20	20	20	20	150
DNA transposons	50						50

[1]Random sequences were generated with the similar length of each MGE type as control

Table S2. Summary of the simulated genomes with different coverages.

Data	LINE[1]	DNA[2]	Satellite	LTR[3]	SVA[4]	Random sequence	# Generated reads(x30)	# Generated reads(x90)
Simulation 1	18	7	3	12	0	20	19,682,260	59,045,715
Simulation 2	12	1	5	20	2	20	19,689,639	59,066,960
Simulation 3	10	1	1	27	1	20	19,685,731	59,055,356
Simulation 4	17	0	3	20	0	20	19,685,079	59,057,831

[1]Non-LTR retrotransposons including LINEs, [2]DNA transposons, [3]LTR retrotransposons, [4]SVA retrotransposons

Table S3. Summarized information of minke whale data and iMGEins results

**Raw data**	MinkeWhale-00.L03.1.fq<br>MinkeWhale-00.L03.2.fq
**Reference**	310752_ref_BalAcu1.0_chrUn
**Mapping tool**	bowtie2(1)
**Alignment rate**	98.53%
**Detected Breakpoints**	765
**Significantly identified insertions**	9

Significantly identified insertions were selected according to the following criteria; Number of unmapped read is 5 or more and the length of contig is 100 or more.

Table S4. PCR sequencing information of 3 randomly selected significant breakpoints.

Loci	Reference contig ID	Start	End	Primer sequence (5'-3')	Primer sequence (3'-5')	Inserted sequence
Loci 1	gi|590091112|ref|NW_006734157.1|	1477	1478	GCACTGATAGTTCCAAACATGTCAG	CAAGTCTGGCTGTGCTATTTGTTAT	ATTTTAGCTTGAAACCTTATCTCAAGAGAATCATATACACTTCACATGAATAAAAATACCTGAACCCCCCCAAAAAAAAAAAAAACCTGAAACCAAACATTTTTAATTGCTCCAATACCCCAAATATAAAGAAAAAATTAAGTCAGAAGACTGGGCAATCTCTATCTCCCCTCCACACTAACCACCCATCCCATGGAAGACCAAGGGAGATCAGACTTTCCAGACTTCTAACACCTGGCTGCTGCAAAATTCTACGGAGACTCCTTGTGGAACAGCAGTCTCCCATCTCTTGTGTCTCTCCCTGTCATGATCATGCAGGA
Loci 2	gi|590096603|ref|NW_006728771.1|	237911	237915	ATGTTGGATAGTTTCAGGTGTACAGC	GAAGTACAGGGTTAGGTTCCTTCG	TTAGGTTGTTACAGGATATTGAGCAGAGTGCCCTGTGCTATAGAGTAGGTCCTTGTTGGTTACCTATTTTAAATATAGCAGGGTGTACATGTCAATCCCCAACTCCCTAACTATCCCTTCCGTCCACCCTTCCCCTCAGTAACCATAAGTTCGTTCTCTAAGTCTGTGAGTCTGTTTCTGACAAATGTTACTTTCTTATTATTTGTGGTAGTTACGTGCTATAAAGTCACCATGGCCACCGAATTA
Loci 3	gi|590100976|ref|NW_006726372.1|	966	966	ATGCACATACTTGCTAAGACGACTG	GAGCTGGCATGAAGACCTCAG	CCAGTGACTTGGAAGGTTTGCAGGAAACCTGAGGAGCGAGATGATGCCATTATCGAGTGAAATGATGTCGTAAATGGGAAGAGCTCAGAGCTGACTTGGACCTGGGGAGGATGTGACTAT

Table S5. Global sequence alignments between sequence found by iMGEins and sequence obtained by PCR experiment using EMBOSS Needle(2).

	Loci 1	Loci 2	Loci 3
**Length**	321	238	120
**Identity**	282/321 (87.9%)	238/238 (100.0%)	120/120 (100.0%)
**Similarity**	282/321 (87.9%)	238/238 (100.0%)	120/120 (100.0%)
**Gaps**	31/321 (9.7%)	0/246 (0.0%)	0/120 (0.0%)
**Score**	1334	1190	600

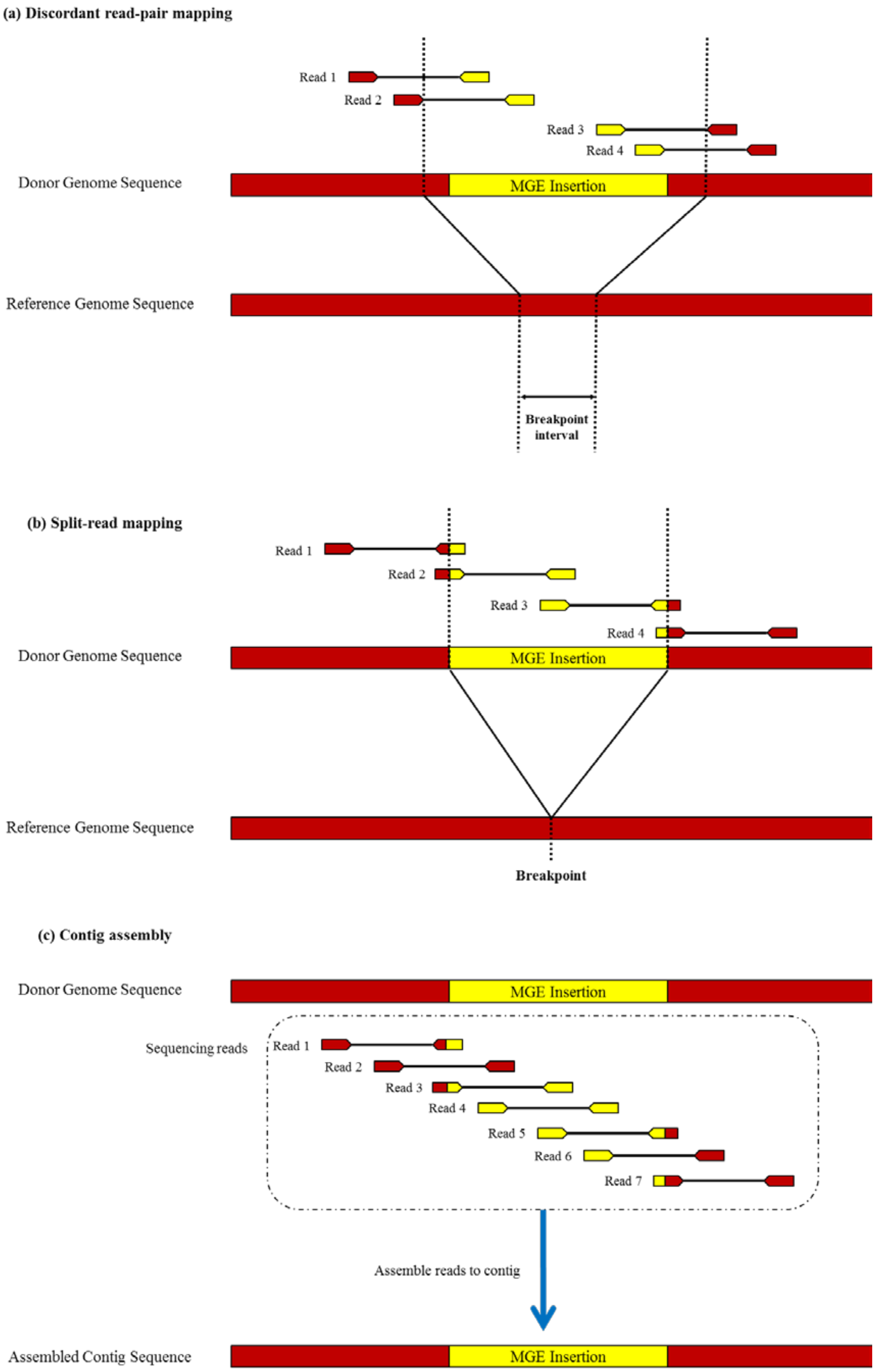

Figure S1. Three approaches of MGE detection algorithm.

(a)

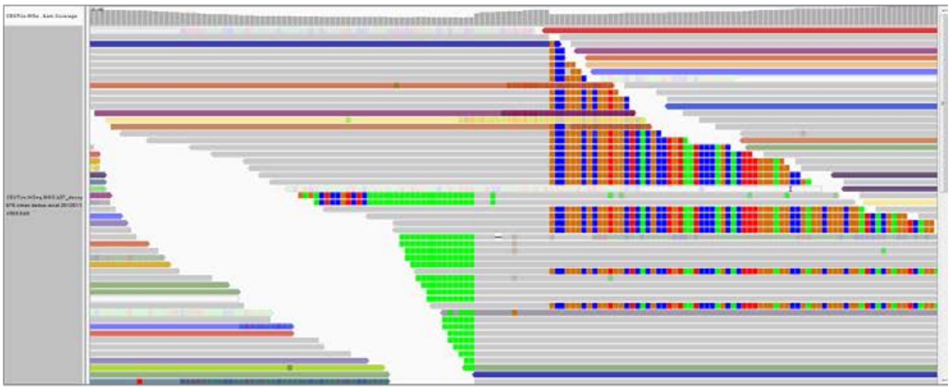

(b)

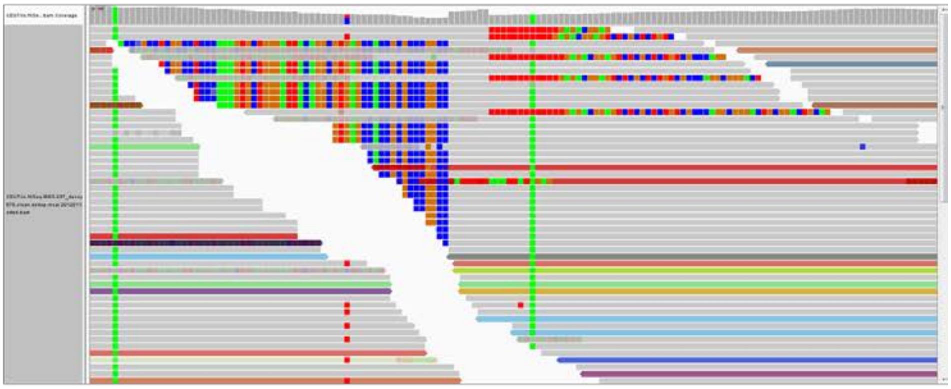

(c)

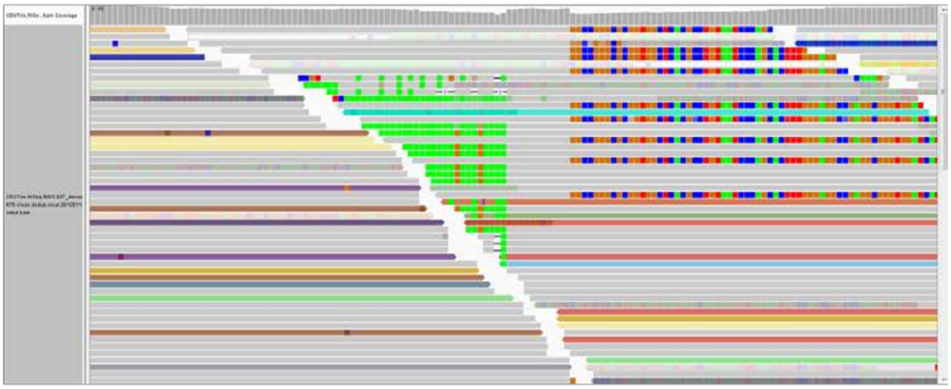

(d)

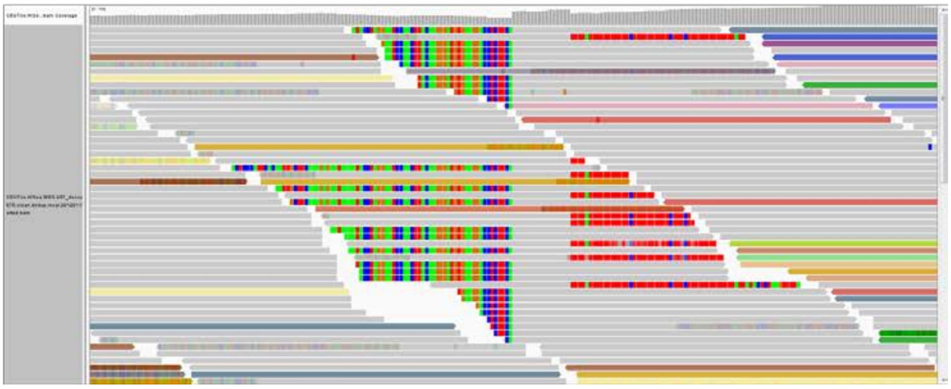

Figure S2. Examples of breakpoints which are not included in the annotation set of NA12878. (a) chr11: 40,494,005, (b) chr1: 79,582,076, (c) chr1: 84,913,265, (d) chr3: 89,022,331.

```
PCR_1             1 TTTTAGCTTGAAACCTTATCTCAAGAGAATCATATACACTTCACATGAAT     50
                    |||||||||.||||||||||||||||||||||||||||||||||||||||
iMGEins_1         1 TTTTAGCTTAAAACCTTATCTCAAGAGAATCATATACACTTCACATGAAT     50

PCR_1            51 AAAAATACCTGAACCCCCCCAAAAAAAAAAAAAAACCTGAAACCAAACATT    100
                    ||||||                           |||||.||.||||||||
iMGEins_1        51 AAAAAT---------------------------ACCTGCAAGCAAACATT     73

PCR_1           101 TTTAATTGCTCCAATACCCCAAATATAAAGAAAAAATTAAGTCAGAAGAC    150
                    |||||||||||||||||||||||||||||||||||.||||.|||||||||
iMGEins_1        74 TTTAATTGCTCCAATACCCCAAATATAAAGAAAAACTTAATTCAGAAGAC    123

PCR_1           151 TGGGCAATCTCTATCTCCCCTCCACACTAACCACCCATCCCATGGAAGAC    200
                    ||||||||||||||||||||||||||||||||||||||||||||||||||
iMGEins_1       124 TGGGCAATCTCTATCTCCCCTCCACACTAACCACCCATCCCATGGAAGAC    173

PCR_1           201 CAAGGGAGATCAGACTTTCCAGACTT---CTAACACCTGGCTGCTGCAAA    247
                    |||||||||||||||.||||||||||   | |||||||||||||||||||
iMGEins_1       174 CAAGGGAGATCAGACCTTCCAGACTTAGGC-AACACCTGGCTGCTGCAAA    222

PCR_1           248 ATTCTACGGAGACTCCTTGTGGAACAGCAGTCTCCCATCTCTTGTGTCTC    297
                    ||||||.||||||||||||||.||||||||||||||||||||||||||||
iMGEins_1       223 ATTCTATGGAGACTCCTTGTGAAACAGCAGTCTCCCATCTCTTGTGTCTC    272

PCR_1           298 TCCCTGTCATGATCATGCAGG     318
                    |||||||||||||||||||||
iMGEins_1       273 TCCCTGTCATGATCATGCAGG     293


PCR_2             1 GTTGTTACAGGATATTGAGCAGAGTGCCCTGTGCTATAGAGTAGGTCCTT     50
                    ||||||||||||||||||||||||||||||||||||||||||||||||||
iMGEins_2         1 GTTGTTACAGGATATTGAGCAGAGTGCCCTGTGCTATAGAGTAGGTCCTT     50

PCR_2            51 GTTGGTTACCTATTTTAAATATAGCAGGGTGTACATGTCAATCCCCAACT    100
                    ||||||||||||||||||||||||||||||||||||||||||||||||||
iMGEins_2        51 GTTGGTTACCTATTTTAAATATAGCAGGGTGTACATGTCAATCCCCAACT    100

PCR_2           101 CCCTAACTATCCCTTCCGTCCACCCTTCCCCTCAGTAACCATAAGTTCGT    150
                    ||||||||||||||||||||||||||||||||||||||||||||||||||
iMGEins_2       101 CCCTAACTATCCCTTCCGTCCACCCTTCCCCTCAGTAACCATAAGTTCGT    150

PCR_2           151 TCTCTAAGTCTGTGAGTCTGTTTCTGACAAATGTTACTTTCTTATTATTT    200
                    ||||||||||||||||||||||||||||||||||||||||||||||||||
iMGEins_2       151 TCTCTAAGTCTGTGAGTCTGTTTCTGACAAATGTTACTTTCTTATTATTT    200

PCR_2           201 GTGGTAGTTACGTGCTATAAAGTCACCATGGCCACCGA     238
                    ||||||||||||||||||||||||||||||||||||||
iMGEins_2       201 GTGGTAGTTACGTGCTATAAAGTCACCATGGCCACCGA     238


PCR_3             1 CCAGTGACTTGGAAGGTTTGCAGGAAACCTGAGGAGCGAGATGATGCCAT     50
                    ||||||||||||||||||||||||||||||||||||||||||||||||||
iMGEins_3         1 CCAGTGACTTGGAAGGTTTGCAGGAAACCTGAGGAGCGAGATGATGCCAT     50

PCR_3            51 TATCGAGTGAAATGATGTCGTAAATGGGAAGAGCTCAGAGCTGACTTGGA    100
                    ||||||||||||||||||||||||||||||||||||||||||||||||||
iMGEins_3        51 TATCGAGTGAAATGATGTCGTAAATGGGAAGAGCTCAGAGCTGACTTGGA    100

PCR_3           101 CCTGGGGAGGATGTGACTAT     120
                    ||||||||||||||||||||
iMGEins_3       101 CCTGGGGAGGATGTGACTAT     120
```

Figure S3. Sequence alignment results for each loci.

References

1. Langmead, B. and Salzberg, S.L. (2012) Fast gapped-read alignment with Bowtie 2. *Nat Methods*, **9**, 357-359.
2. Li, W., Cowley, A., Uludag, M., Gur, T., McWilliam, H., Squizzato, S., Park, Y.M., Buso, N. and Lopez, R. (2015) The EMBL-EBI bioinformatics web and programmatic tools framework. *Nucleic Acids Res*, **43**, W580-584.
